# Photo Quiz: An unexpected colon companion

**DOI:** 10.1128/jcm.01331-25

**Published:** 2026-05-04

**Authors:** Aleksander Luniewski, Jacob Kaplan, Melinda D. Poulter, Emily A. Snavely

**Affiliations:** 1 Division of Laboratory Medicine, Department of Pathology, University of Virginia School of Medicine 189557, Charlottesville, Virginia, USA; Mayo Clinic Minnesota, Rochester, Minnesota, USA

**Keywords:** parasitology, Helminth

## PHOTO QUIZ 

**Fig 1:**
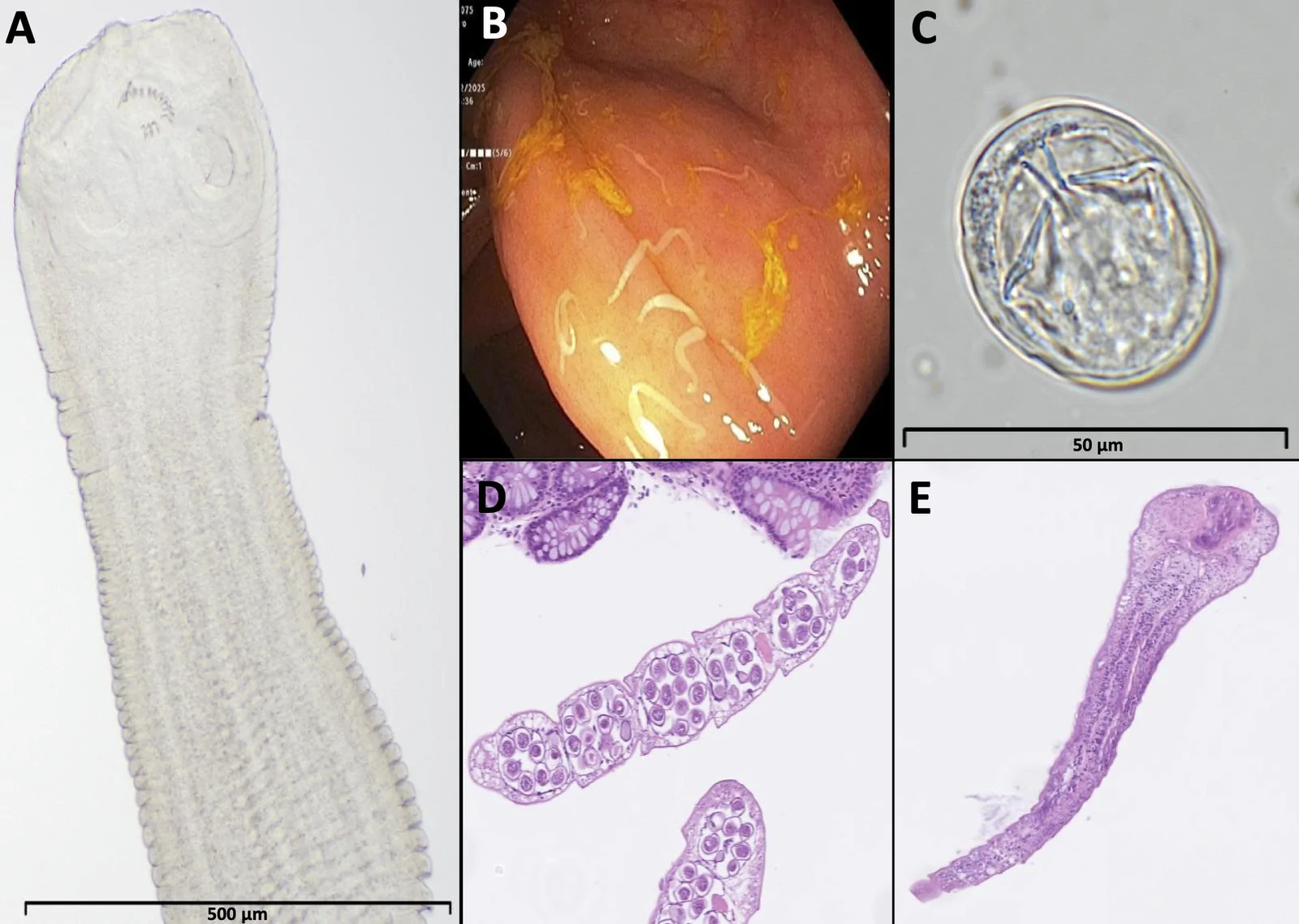
Morphologic features of *Rodentolepis nana*. (**A**) Light microscopy of an adult worm. (**B**) Colonoscopic image showing multiple worms within the colonic lumen. (**C**) Light microscopy of a *R. nana* oncosphere liberated from an egg. (**D**) Hematoxylin and eosin (H&E) section demonstrating overlapping proglottids. (**E**) H&E section highlighting the scolex.

A 51-year-old male with no significant past medical history presented to an academic medical center for a routine high-risk colon cancer surveillance colonoscopy. His previous colonoscopy, performed 4 years earlier, revealed five small adenomas and a sessile serrated polyp, but no other abnormalities were noted. During the current examination, two additional tubular adenomas and one sessile serrated polyp without dysplasia were identified. In addition, multiple elongated worms were unexpectedly visualized in the descending colon, ascending colon, and cecum (Fig. 1B). The worms appeared flat on the endoscopic imaging and were observed lying along the mucosal surface. Otherwise, the colonic mucosa was without abnormality. Representative specimens were biopsied with cold forceps and submitted for further workup. Whole worms were sent to the clinical microbiology laboratory for examination under light microscopy (Fig. 1A and C), while biopsy fragments of colonic mucosa containing portions of the parasite were submitted for histological evaluation by surgical pathology using hematoxylin and eosin staining (Fig. 1D and E).

At a follow-up appointment, the patient reported some abdominal discomfort, fatigue, intermittent mild headaches, and intermittent anal pruritus. No recent travel history, unusual dietary exposures, or animal contacts were noted, although these were not specifically elicited. No additional stool studies or serologic testing were pursued following the colonoscopy findings.

## ANSWER TO PHOTO QUIZ

*Rodentolepsis nana*, or “dwarf tapeworm,” is the most common human cestode infection worldwide (1). Transmission can occur through the ingestion of embryonated eggs on contaminated food, water, or hands (2). The eggs are immediately infectious when passed in stool but survive in the environment for about 10 days (3). Unlike other cestodes, *R. nana* can complete its life cycle without requiring an intermediate host, which contributes to its high prevalence compared to *Hymenolepis diminuta*. Additionally, arthropods such as beetles can serve as intermediate hosts (3, 4).

Infection begins when ingested eggs release oncospheres in the small intestine (1, 2). These penetrate the villi and develop into cysticercoid larvae, which later re-enter the lumen, attach to the mucosa, and mature into adult worms in ~4 weeks (1, 3). Adults, which typically reside in the ileum, live only for 4–6 weeks (1, 3). Autoinfection occurs when eggs hatch *in situ* and re-invade the mucosa, allowing infection to persist for years despite the short lifespan of individual worms (3).

Diagnosis relies on microscopic examination of stool for ova and proglottids. Eggs of *R. nana* are oval, 30–50 µm, with a thin shell, an oncosphere containing six hooklets, and 4–8 polar filaments extending between the membranes. In this case, microscopy demonstrated an oncosphere separated from the egg, with characteristic hooklets (Fig. 1C). By contrast, *H. diminuta* eggs are larger (70–85 × 60–80 µm), possess a striated outer membrane, and lack polar filaments (3). Adult *R. nana* worms measure 20–45 mm by 1 mm, with a scolex bearing four suckers and an armed rostellum (Fig. 1A). *H. diminuta* adults are substantially larger (20–60 cm) and have a scolex with four suckers and an unarmed rostellum (5). Craspedote proglottids, which overlap in series, are visible by light microscopy or histology (Fig. 1A and D).

Children and populations in crowded or low-sanitation settings are at greatest risk (1, 3). Infections are often asymptomatic, but in heavy worm burdens, patients may present with diarrhea, abdominal pain, headache, anorexia, and pruritus ani (1, 3). Praziquantel is the treatment of choice, although re-infection is common without improved hygiene and sanitation (1). Our patient was prescribed praziquantel with no reported complications following treatment.
